# Strategies identified by program directors to improve adoption of the CanMEDS framework

**Published:** 2018-11-12

**Authors:** Isabelle Gaboury, Kathleen Ouellet, Marianne Xhignesse, Christina St-Onge

**Affiliations:** 1 Department of Family Medicine and Emergency Medicine, Faculty of Medicine and Health Sciences, Université de Sherbrooke, Quebec, Canada; 2 Center for Health Profession Education, Faculty of Medicine and Health Sciences, Université de Sherbrooke, Quebec, Canada; 3 Department of Medicine, Faculty of Medicine and Health Sciences, Université de Sherbrooke, Quebec, Canada

## Abstract

**Background:**

Challenges associated with the use of the CanMEDS physician competency framework (CanMEDS) have been the subject of several studies. Most of these have focused on the adoption of specific roles in an Anglophone context. This study aims to investigate how Francophone postgraduate medical education (PGME) program directors have integrated the CanMEDS framework into their programs.

**Methods:**

We invited Francophone PGME program directors to participate in group interviews aimed at exploring their experiences using the CanMEDS framework. We used an open-ended interview guide and realized a thematic analysis of the transcripts.

**Results:**

We held five group interviews between February and December 2014 with 17 Francophone program directors representing 13 out of a maximum of 62 different specialties/subspecialties. Although program directors endorsed the framework, its integration was seen as challenging, particularly the assessment of non-medical expert roles. To overcome these challenges, they relied on common strategies including a longitudinal approach to the framework, improving inter-program collaboration, and subcontracting the teaching of certain roles.

**Conclusion:**

While integrating the CanMEDS framework into their programs, Francophone program directors struggled with teaching and assessing non-medical expert roles and ensuring their longitudinal integration over time. Directors relied on various strategies, some of which (e.g., subcontracting) may ultimately limit the adoption of the framework as a whole. ___

## Abstract (fr)

**Contexte:**

Les défis associés à l’utilisation du référentiel de compétences CanMEDS pour les médecins ont fait l’objet de plusieurs études. La plupart de ces études ont portées sur l’adoption de rôles spécifiques dans un contexte anglophone. Cette étude vise à explorer comment les directeurs de programmes d’études médicales postdoctorales (EMP) francophones ont intégré CanMEDS dans leurs programmes.

**Méthodes:**

Nous avons invité les directeurs de programmes EMP francophones à participer à des entrevues de groupe. Ces entrevues visaient à explorer leur expérience de l’utilisation du référentiel CanMEDS. Nous avons utilisé un guide d’entrevue ouvert et nous avons fait une analyse thématique des transcriptions.

**Résultats:**

Nous avons tenu cinq entrevues de groupe entre février et décembre 2014 avec 17 directeurs de programmes de 13 des 62 spécialités/sous-spécialités. Bien que les directeurs de programmes appuient le référentiel, son intégration a été perçue comme un défi, notamment en ce qui a trait à l’évaluation des rôles autres que celui d’expert médical. Pour surmonter ces défis, ils se sont appuyés sur des stratégies communes, notamment une approche longitudinale du référentiel, l’amélioration de la collaboration entre les programmes et la sous-traitance de l’enseignement de certains rôles.

**Conclusions:**

À travers le processus d’intégration du référentiel CanMEDS, les directeurs de programmes EMP francophones ont de la difficulté à enseigner et à évaluer les rôles autres que celui d’expert médical ainsi qu’à veiller à leur intégration respective et continue au fil du temps. Ils ont eu recours à diverses stratégies, dont certaines (p. ex., la sous-traitance) pourraient ultimement limiter l’adoption du référentiel dans son ensemble.

## Introduction

There has been an increased interest in competency-based education (CBE) in recent years, with many different CBE frameworks published in the health professions education (HPE) literature.^1–5^ For this study, we adopted the definition proposed by Frank et al.: “Competency-based education (CBE) is an approach to preparing physicians for practice that is fundamentally oriented to graduate outcome abilities and organized around competencies derived from an analysis of societal and patient needs. It de-emphasizes time-based training and promises greater accountability, flexibility, and learner-centredness”(p.636).^6^ CBE and CBE-based frameworks were developed in response to pragmatic pedagogical and societal needs^7,8^ to appropriately address society’s health care needs.^6,9^ The Royal College of Physicians and Surgeons of Canada (RCPSC) CanMEDS physician competency framework^10^ is one example of CBE being used around the world,^11–13^ in postgraduate medical education as well as undergraduate medical education programs.^14^ This framework is bilingual and it is used in both Francophone and Anglophone programs. The 2005 version included seven roles, namely, “medical expert,” “communicator,” “collaborator,” “scholar,” “manager,” “health advocate,” and “professional.” In the 2015 version, the “manager” role was renamed “leader” and redefined.

Many scholars have critiqued various competency frameworks suggesting that defining competence through a series of roles might minimize the concept of competence and not appropriately reflect its complexity.^15–17^ Pragmatic issues associated with the implementation of CBE-related frameworks have also emerged. Authors have called for increased alignment of the frameworks with specialty-specific considerations.^18,19^ Independent of specialty, it appears that the adoption of the CanMEDS framework requires some adaptation by its main users – program directors, administrators, and clinical teachers – in order to make implementation sustainable. Despite the publication of a CanMEDS implementation strategy^11^ many scholars have shown that integrating individual roles into training programs can be challenging, especially when it comes to the assessment of the so called “intrinsic roles” (i.e., non-medical expert CanMEDS roles).^12,20–26^ Moreover, reports of the challenges associated with use of the CanMEDS framework have focused on individual roles, distinct from adoption of the framework as a whole.

In spite of its continuous evolution, arrival of the revised 2015 CanMEDS framework^27^ and efforts to be consistent with the implementation of “Competence by Design” (CBD) as defined by the RCPSC, have created an impetus for the investigation of the adoption of the CanMEDS competency framework as a whole. According to Rogers’s Diffusion of Innovations Theory, adoption refers to the decision of “full use of an innovation as the best course of action available” (p.177).^28^ A core principle of CBD is that competencies are acquired on a continuum. To our knowledge, Puddester et al.^20^ and Whitehead et al.^29^ are the only authors who have tried to investigate faculty members’ needs with respect to teaching and assessing non-medical expert roles in this manner. Still, little is known about the *processes* associated with the overall adoption of the CanMEDS framework by its users, whether in Francophone or Anglophone environments. Moreover, since perceptions and practices vary with culture, adoption of a framework could potentially vary across different contexts. According to Kramsch,^30^ language is a vehicle of thought and culture. Although the CanMEDS framework was rolled out in Canada in both English and French, its adoption overall has not been well studied in a Francophone context.

Given these gaps, we conducted an exploratory study to investigate how Francophone postgraduate medical education (PGME) program directors in different faculties adopted and integrated the CanMEDS framework into their programs. More specifically, we conducted group interviews to identify specific challenges underlying the use of the CanMEDS framework, and strategies used to address these challenges in a Francophone context.

## Methods

### Study Design

In an effort to better understand the adoption of the CanMEDS framework in a Francophone context, all PGME program directors from all specialties of the four Quebec faculties of medicine were approached through their PGME deans to participate in group interviews. The number of specialties/subspecialties represented varies between faculties, but there are a maximum of 62 specialties/subspecialties educated in Quebec.^31^ Group interviews were preferred to individual interviews in order to facilitate and encourage inter-participant exchanges and to highlight program similarities. Informed consent was obtained from participants and group interviews were conducted in French by two co-authors (IG and KO), who had no connection with the program directors who were interviewed. We used a semi-structured interview to explore program directors’ experiences using the CanMEDS framework as a tool to guide curriculum development and teaching, as well as the assessment of medical competencies. The interview guide was loosely inspired by Rogers’s Diffusion of Innovations Theory^28^ where the relative advantage, compatibility and perceived complexity of the CanMEDS framework, as well as the main drivers of adoption of the framework for curriculum development, teaching, and assessment, were the focus of the discussion. The interview guide (process and questions), which is presented in Table 1, was tested with three former PGME program directors to ensure question clarity and relevance. Group discussions were audiotaped and transcribed verbatim. We obtained ethical approval from the Research Ethics Board – Education and Social Science, Université de Sherbrooke.

**Table 1:** Interview guide: process and questions

1. Participants were first asked “**As a program director, what are your thoughts regarding the usefulness of the CanMEDS framework** (relative advantage)?”
2. We then entered into specific use of the framework in their own context, by asking them to **highlight the perceived benefits or difficulties of using the framework** (compatibility and complexity).
3. In order to explore the adoption of the framework, participants were asked **to illustrate their points by examples,** but were reminded to think of the framework as a whole rather than limiting their reflection to specific roles in their examples.
4. Finally, we concluded the group interviews by asking participants if there was **anything undiscussed about the CanMEDS framework** that they would like to discuss.

### Data Analysis

We read and analyzed the transcripts in a stepwise manner using an iterative process to interpret the data^32–34^ based on the conceptual framework chosen for the interview guide. First, verbatim transcripts were read and coding categories were created using NVivo software Version 9.0 (QRS International Inc., Burlington, MA, USA). These codes were then used to categorize excerpts of the transcripts until no new categories emerged. Coding and analysis were conducted independently by two co-authors (IG and KO) and any disagreements were discussed until a consensus was reached. The prominent codes were then extracted and only the themes that reached saturation were preserved; that is, themes for which sufficient data were collected to allow comprehension of a concept.^35^ The overall analysis was discussed regularly among all authors during and after the group interviews to improve the rigor of the analysis and the credibility of the findings. The whole process was conducted in French and only the excerpts presented were translated into English.

## Results

In total, 17 residency program directors from two Francophone faculties of medicine participated in the study, representing 13 different specialties/subspecialties. Group interviews were scheduled by faculty and availability of participants guided the composition of each group. Whenever possible, efforts were made to vary the composition of each group in terms of specialities/subspecialties to enrich interview discussions. Five group interviews (between two and five participants per group) took place between February and December 2014, and discussions lasted on average for 51 minutes. Table 2 presents the main characteristics of participants.

**Table 2:** Main characteristics of the 17 participants

	**Participants (n)**
**Gender**	
Male	8
Female	9
Specialty^*^	
General specialty	7
Subspecialty	10

* Classification of the specialties is based on that of the Royal College of Physicians and Surgeons of Canada (RCPSC)

Results are organized according to the two main findings of the study: 1) program directors’ endorsement of the CanMEDS framework (namely, CanMEDS 2005); and 2) challenges underlying the adoption of the CanMEDS framework and common strategies to address these.

### Program directors’ endorsement of the CanMEDS framework

According to the CanMEDS 2005 framework, being a good physician requires integrating seven roles. The participants’ reported that the CanMEDS framework helps train residents as it guides them to develop competencies in addition to those of the medical expert. In doing so, they thought the CanMEDS framework was contributing to the training of more “complete” physicians: “For future generations, I think it makes for more competent, more all-round professionals, when they really have [acquired] all the CanMEDS abilities.” (Participant 2, Group 2)

Giving formal expression to what was previously informal seemingly reinforced to participants the merits of adhering to the CanMEDS framework: “I find that it helps to put into words realities that have always been there. This is beneficial.” (Participant 1, Group 4)

According to participants, the framework enables stakeholders to speak a common language; the seven competencies are “dissected,” allowing some standardization of the vocabulary used to describe what is expected of residents. Competencies form the common structure on which residents are assessed. Moreover, breaking down expectations into various competencies was seen as allowing for better organization of the different facets to be developed.

For most participants, the division into seven roles allows for more targeted interventions. Additionally, the clearly identified and defined roles were seen as assisting in addressing the expected competencies within each role.

*It’s much simpler now when we meet* [with residents to assess them], *first, because we have a better understanding of the different roles, so we’re in a much better position to say, “Look, for this one competency, you’re doing very well.” For all of them, except for that one (…).” It’s a lot simpler now because it’s divided up, and I find it’s easier to take action*. (Participant 2, Group 3)

### Challenges underlying the adoption of the CanMEDS framework and common strategies to address these

Despite adhering to the principles of the CanMEDS competency framework and the perceived benefits of proposing a common structure, participants reported several challenges, most of which were related to the operationalization of the framework. Strategies used by participants to minimize the impacts of these challenges within their programs were identified throughout the interviews.

While the program directors who we met appeared to navigate easily through the process of teaching the clinical skills of the medical expert, the six non-medical expert CanMEDS roles (communicator, professional, manager (leader in CanMEDS 2015), collaborator, health advocate, and scholar) posed greater challenges. Participants criticized the perceived lack of training available on how to teach competencies other than those of the medical expert. Most participants admitted feeling ill-equipped to teach these other competencies, regardless of whether their medical training took place prior to or after their introduction to a CanMEDS competency framework.

*The difficulty [for us] as program directors is that we’re used to training by role models. A good example is the example that residents will give. We’re well equipped to give technical training; we know how to do that. What’s harder, at least from our perspective, in terms of introducing the CanMEDS roles, is the lack of role models, plus the fact that we have to be creative*. (Participant 1, Group 3)

Participants also expressed the feeling that they have little support for teaching/assessing competencies other than those of the medical expert. They find the requirements for these vague and unclear, which obliges them to “create” their own teaching/assessment tools.

*We had to invent [our own 360° assessment form] because none really exists. How can it be that there is none? How can it be that we’re expected to do that? There are no tools. That’s what’s missing*. (Participant 2, Group 2)

Some participants also reported that they felt disadvantaged because many tools were created by other programs (instead of the RCPSC), and consequently, were not available in French.

We [PGME program directors] help each other, we share documents: « Here is how I assess this part… » (…) But when they [Anglophone PGME program directors] send me their documents, I take what I want, but I have to do the translation. (Participant 2, Group 3)

A handful of strategies to improve adoption of the CanMEDS competency framework are emerging in the face of these challenges. The first translates into a more longitudinal approach to the framework. While the framework reflects the RCPSC requirements, participants see it more like an “integrating” structure, as described in the following comment:

I realize that sometimes, for certain CanMEDS competencies, rather than dividing them up by rotations, it would be better to have something a little more longitudinal; to really see the resident as a whole. (Participant 4, Group 2)

A second approach to improving the framework’s adoption was inter-program collaboration. The creation of opportunities to share experiences with colleagues in other programs or to develop common inter-program activities, both appear to be worthwhile initiatives as new implementation strategies can emerge from these collaborations. While major differences sometimes exist between programs, these strategies allow inspiration to be drawn from other people’s work and boost the efforts within a given program to teach and assess the competencies.

The creation of working groups with directors of other programs (internally) or with other medical faculties offering similar programs fits this approach to improve adoption. As expressed by the following participant, these collaborations allow program directors to open their minds to new ways of doing things:

*Sometimes there are competencies where you say to yourself, “But we don’t cover this competency. How could we cover it?” So it might be helpful if we could work with other programs, precisely because that would give us more ideas than if we’re just focused on ourselves and turning around in circles because we don’t know how to go about teaching this competency*. (Participant 1, Group 5)

A third strategy emerged from the difficulty of teaching/assessing competencies other than those of the medical expert: the strategy of subcontracting the teaching to professionals “specialized” in the different roles. This strategy poses major difficulties – efforts to do so can be fruitless because “specialists” are rare. While some individuals may have developed a network over the years allowing them to delegate the teaching of certain competencies to various “experts,” others may not, thus making it a challenge to secure these experts. In this respect, all the participants regard the assessment of the CanMEDS competencies associated with the non-medical expert roles as a challenge, in that they see the assessment requirements as vague and non-prescriptive, which obliges them to “fumble their way along.”

*I find the manager role very difficult. I turn to the administrative director of our department and ask him to talk with the residents about all aspects of the department’s operations, like purchasing, upgrading equipment, and how we go about hiring professionals. Because I don’t know anything about that. And then there’s assessment, which is not as simple as that. In a case like that, I’d have to ask him to give a test, but he has already agreed to give the course…* (Participant 2, Group 4)

## Discussion

The relative advantages and compatibility of the CanMEDS framework and CBE with the participants’ needs is recognized, but Quebec Francophone residency program directors perceive the framework to be difficult to implement. Guided by Rogers’ Diffusion of Innovation Theory, five group interviews involving 17 program directors from two Quebec medical schools, revealed strategies in response to implementation challenges, particularly for the non-medical expert roles.

### Challenges underlying the adoption of the CanMEDS framework

Some difficulties may be experienced in shifting from a theoretical framework to practice: indeed, the challenges reported by our participants concern the difficulty of operationalizing the CanMEDS framework and even more with integrating the different roles over the duration of the training. This finding adds to the results of Whitehead *et al*.^29^ who reported, among other things, that program directors perceive themselves as lacking the expertise needed to teach and assess the non-medical expert roles. Puddester et al.^20^ focused more on competency assessment, also showing professors’ need for training in the assessment of the non-medical expert roles, which remains a disintegrated vision of the CanMEDS framework. Nonetheless, the challenge of operationalization is present in both Anglophone and Francophone contexts. The non-medical expert roles are seen as difficult to teach, and equally difficult (if not more so) to assess.

For Rogers,^28^ incentives may increase motivation, resulting in a more effective adoption of an innovation. Contrary to Puddester et al.’s study,^20^ none of our participants mentioned the lack of any kind of incentives (whether financial or linked to a career advantage) as a barrier to implementation of teaching the competencies of the CanMEDS framework. This is also contrary to what has been reported in the context of other competency frameworks like the Accreditation Council for Graduate Medical Education (ACGME) framework.^36^

### Strategies to address the challenges

We sought to move beyond the observation that “challenges” exist in the teaching and assessment of CanMEDS roles by bringing to the forefront strategies proposed by program directors to address these challenges. Our participants’ proposals echoed the results obtained by Puddester et al.^20^ who, through an analysis of program directors’ needs in a single faculty of medicine (English-speaking context), identified the need for collaboration and a sharing of resources. Our participants believe that the CanMEDS roles should be taught and assessed in a more longitudinal and collaborative manner. Although having a longitudinal perspective on development may seem self-evident in a competency framework, its manifestation did not appear that simple for our participants. They reported struggling to implement the framework and often needed to break it down into smaller parts; however, this tactic runs the inherent risk of losing sight of the overall and developmental objective of the framework.

The 2015 version of the CanMEDS competency framework appears to be congruent with these strategies: the framework integrates several competency milestones associated with an acquired and validated professional activity.^27^ The act of explicitly integrating several competency milestones, however, further fragments the roles. Residents are thus assessed in terms of a range of sub-competencies. In this context, and given the already overloaded curricula, the strategies proposed by our participants seem all the more pertinent. The challenge of “not reinventing the wheel,” by avoiding working in isolation,^29^ therefore still stands.

The strategy of subcontracting the teaching of the non-medical expert roles to “specialized” professionals may align with more longitudinal teaching and assessment, and with improved collaboration however, it may also, limit adoption of the framework as a whole. The fact remains, however, that this strategy highlights the program directors’ unease regarding the best ways to proceed teaching and assessing non-medical expert competencies. As underscored by Mickelson,^37^ this unease may be due to “a lack of understanding of what the roles actually represent” (p. 395). Competency assessment remains a problem when competencies are taught by one group of individuals but assessed by another. This perception of not having the expertise required to teach the non-medical expert roles may also mean that program directors do not capitalize on role modeling, even though this strategy is generally recognized as important.^38^ It could be interesting to explore “train-the-trainer” approaches for this problem so that a broader range of faculty consider themselves more comfortable modeling, teaching, and assessing the non-medical expert roles. In addition, the longitudinal integration of the non-medical expert roles over the duration of training could be facilitated by a model more closely connected with assessment.

### Conclusion

The goal of this study was to explore Francophone PGME program directors’ adoption of the CanMEDS 2005 framework as a whole. Our results suggest that despite the presence of several challenges related to the operationalization of roles, Francophone program directors supported the concept of CBE and the CanMEDS framework. They relied on various strategies to overcome challenges encountered, but they struggled, just as Anglophone directors, with teaching and assessing the non-medical expert roles to ensure their longitudinal integration over time. In addition, some of the strategies used (e.g., subcontracting) may in fact have the untoward effect of limiting adoption of the framework as a whole.

This study has some limitations. First, the fact that participants were from two of the four faculties of medicine in Quebec may limit the transferability of our results. Indeed, it is possible that Francophone participants from other faculties might have a different point of view. The composition of each interview group may also have influenced the findings obtained. Additionally, we cannot rule out that program directors who volunteered for the group interviews might have had a bias (be it positive or negative) towards the CanMEDS framework and its use in the curriculum.^39^

While participants called for more guidance on how to teach and assess the non-medical expert roles, the solution may not lie in the sharing of resources. In fact, despite the CanMEDS implementation strategies made available by the RCPSC, program directors still struggle with teaching, as well as assessing, non-medical expert roles. A collaborative approach to finding true solutions is needed to do away with *ad hoc* Band-Aid solutions. A theory-based evaluation of the implementation of CanMEDS, in partnership with program directors from different cultural contexts may optimize adoption in the future and in turn improve curriculum development, teaching, and assessment. Replication of this project in countries other than Canada where the CanMEDS framework is used may also offer a broader perspective on the local and cultural difficulties and solutions related to the implementation of the framework.
